# Prevalence of Dangerous Ethmoid in a Tertiary Center in Eastern Nepal

**DOI:** 10.31729/jnma.4637

**Published:** 2019-10-31

**Authors:** Sudeep Mishra, Shyam Thapa Chhetri, Ashok Raj Pant, Shankar Prasad Sah, Sriti Manandhar

**Affiliations:** 1 Department of Otolaryngology, BP Koirala Institute of Health Sciences, Dharan, Nepal; 2 Department of Radio Diagnosis, BP Koirala Institute of Health Sciences, Dharan, Nepal

**Keywords:** *computed tomography*, *endoscopy*, *ethmoid sinus*

## Abstract

**Introduction:**

Endoscopic sinus surgery is a well-known approach for sino-nasal pathologies. Due to close proximity to the brain and orbit, the surgeon should be aware of the sino-nasal anatomy and its associated variations. Detailed preoperative assessment of the sinus computed tomography scans reduces the frequency of severe complications in patients undergoing an endoscopic sinus surgery. So, the aim of this study is to find the prevalence of dangerous ethmoid in a tertiary center in eastern Nepal.

**Methods:**

A descriptive cross-sectional study was performed in a Computed tomography scan of 50 patients with chronic sinusitis undergoing endoscopic sinus surgery from February 2018 to August 2018 in the department of Otolaryngology and Radiology of BP Koirala institute of health sciences after taking ethical approval from Institutional Review Committee of the institute. Measurements are taken in the coronal plane. The depth of the lateral lamella of the cribriform plate was defined according to the Keros classification which defines the dangerous ethmoid. and side. Data entered in MS excel. Statistical analysis was performed in SPSS version 11.5.

**Results:**

Keros type I, II, and III were noted in 17 (17%), 54 (54%) and 29 (29%) of cases respectively. The mean width of the olfactory fossa, medial orbital wall distance and distance from medial nasal concha were 3.57mm, 8.77mm & 17.78mm respectively.

**Conclusions:**

The most common type of dangerous ethmoid was keros type II.

## INTRODUCTION

The most commonly injured part in endoscopic sinus surgery is the lateral lamella of the cribriform plate.^1^ CT reveals the variations and mucosal pathology.^2-4^ The anatomical variations play a very important role in the pathogenesis of the sinonasal region.^5^

Keros classified the olfactory fossa depth into Type I (1-3mm), Type II (4-7mm), and Type III (8-16mm).^6^ The type III fossa is weak and is less protected. Kainz, et al. coined the term dangerous ethmoid, based on the depth of the olfactory fossa. The other features of the roof of the nasal cavity have not been evaluated thoroughly, which include the position of the anterior ethmoid artery and the position of the lateral lamella in relation to the cribriform plate.^7^

The findings of the anterior ethmoid complex will caution the surgeon and will reduce the chance of major complications.^8-10^

The aim of this study is to find the prevalence of dangerous ethmoid in a tertiary center in eastern Nepal.

## METHODS

A descriptive cross-sectional study was conducted from February to August of 2018 in the department of Otolaryngology and Radiology among adults of 18 years and above diagnosed with chronic sinusitis undergoing CT before surgery. Ethical approval was taken from the institutional review committee. Patients with a history of trauma, malignancy, and history of previous surgery were excluded from the study.

The sample size was calculated by convienient samoling method using a 95% Confidence Interval. For this purpose, the study considers the common occurrence of Keros II percentage in the literature as 73.28%.

Now using the following formula,

Sample size calculation:

Using Z= 1.96 at 95% Cl
p= Prevalence, 73.28% (educated guess)
q=1-p, 26.72
e= margin of error = 14.6%

Now, sample size is given by,

n = Z^2^ × p × q/e^2^

   = (1.96)^2^ × 73.28 × 26.72)/(14.6)^2^

   =35.288≈36

However, all the 100 cases in 50 patients were taken which meet the inclusion criteria.

Measurements were taken during the CT scan in a coronal plane. Performa was filled up. Data entered in MS excel. Statistical analysis was performed in SPSS version 11.5. Descriptive statistics were presented as percentage, mean, median, range, interquartile range, SD, in tabular and graphical representation.

## RESULTS

The majority of the patients in this sample had a type II olfactory fossa 54 (54%), whereas type I and type III were recorded in 17 (17%) and 29 (29%) respectively (Table 1).

**Table 1:** Prevalence of Dangerous Ethmoid according to Keros Classification.

Types	n (%)
Type I	17(17%)
Type II	54 (54%)
Type III	29 (29%)

For further analysis, the parameters on the left and right sides were regarded as separate cases, so 100 cases in 50 patients were analyzed.

The average patient age was 39.4 years. Out of them, 32 (64%) were male and 18 (36%) were females. The depth of the olfactory fossa was 6.8±2.8, 1.9-17.4.

The findings of the width of the olfactory, distance from Cribiform Plate to orbit and distance from Cribiform Plate to Middle Turbinate are summarized (Table 2).

**Table 2:** Comparison of means of measured width of Cribiform Plate, Length of Middle Turbinate & Orbital distance between the left and right sides.

Measurements (mm)	Mean (Right)	Mean (Left)	SD (Right)	SD (Left)
Width of the olfactory fossa	3.578	3.890	2.6	2.2
Length of the middle turbinate	17.712	17.866	6.0	6.1
Distance between the orbit and olfactory fossa	8.522	9.036	3.6	4.0

These values were higher on the left side. The means of these measurements were compared between the male and female gender. The mean distance measured between the widths, length of middle turbinate and orbital distance were higher in males than females (Table 3).

**Table 3:** Comparison of means of measured width of CP, Length of MT & orbital distance between both genders.

Measurements (mm)	Female number Right/Left	Male Right/Left
Width of the olfactory fossa	3.411/3.778	3.672/3.953
Length of the middle turbinate	17.111/17.550	18.050/18.044
Distance between the orbit and olfactory fossa	8.183/8.844	8.712/9.144

## DISCUSSION

The paranasal sinus exhibit complex anatomical variations and it is very important for endoscopic sinus surgery. CT scan evaluation reveals the anatomical details and especially the thin section is important for the identification of normal anatomy, variations, and diseases. The coronal plane is the best to visualize the ethmoid roof anatomy.

The majority of patients have Keros type II olfactory fossa depth 54% whereas Keros type I and Keros III were 17% & 29% respectively. Our findings were similar to Virgo et al. classified Keros II 58%, type I 14% & type III 28%. Similar findings were recorded by Nitinavakarn et al. type I 11.9%, type II 68.8%, & type III 19.3%, and Erdem et al. type I 8.1%, type II 58.6% & type III 32.3% which were similar to those reported in the literature.^12,13^

The median depth of the olfactory fossa was 6.8 mm. These findings were consistent with Basak et al. which was 5.99±2.3 mm.^14^

Type III is most dangerous for the surgeon because of the likelihood of perforation through the lateral lamella of the cribriform plate.^15^ The deeper the lamina cribrosa, the thinner the lateral lamella.^16^

The preoperative radiological imaging of patients with the pathological process must help in assessing the disease-related changes as well as the normal anatomical landmark increasing the risk of iatrogenic complications. The presence of natural bony loss (dehiscence) and/or rarefaction may predispose to iatrogenic damage as these may weaken the bony structures and these occurrence increases as the depth of the olfactory fossa increases. These bony defects were observed in 53% of cases with chronic sinusitis with keros type I and 69.5% in Keros type II olfactory fossa depth.^16^ The use of navigation systems has increased the safety of such procedures preventing and minimizing the risk of injury. Our results showed neither age, sex, side of the body or the type of Keros classification facilitate the high-risk group.

Different studies used different radiological parameters. Previously only the olfactory fossa depth was considered to be an important parameter for dangerous ethmoid, however other parameters should also need to be considered. Andrzej Skorek, et al. considered additional anatomical parameters to equally important to evaluate.^17^

These include:

1) The width of the olfactory fossa.

2) The distance from the olfactory fossa to the medial concha.

3) The distance from the olfactory fossa to the medial wall of the orbit.

The critical measurement, in addition to Keros classification, should be considered are width of the olfactory fossa <10mm, width of the olfactory fossa >6mm and length of the middle turbinate <20mm, which classify the potential risk for skull base or lamellar injury.^17^ In our study, the width of the olfactory fossa>6mm was seen in 10 out of 100 slides, should be regarded as a red flag as a possible dangerous ethmoid (Fig 6).

The anterior cranial fossa boundary comprising of ethmoid roof and orbit, defining the superior and lateral limit of the surgical field. The middle nasal concha is a consistent and important landmark during endoscopic sinus surgery as it forms the lateral boundary and its presence to prevent iatrogenic injuries, however in cases of revision surgery, the anatomical landmarks are lost because of the disease posing a risk for injury. There are very few studies that measured the length of middle turbinate for asymmetry and variations and its relation with the cribriform plate. Ahmet Songur, et al. in his study of 300 cases measured the depth and anatomical variations of the ethmoid roof, found the average vertical length of the middle turbinate was 23.7mm on Right and 24.34mm on the left side.^18^ The distance between the ethmoid roof and the nasal oor was largest in Keros type III.^17^ These critical measurements can be performed on Routine CT evaluation without the need for any software. Further attention should also be paid to the asymmetry of cribriform plate/lateral lamella. These above-mentioned findings should be considered as dangerous ethmoid during the preoperative CT scan evaluation. Due to high asymmetry, further retrospective and prospective study need to be carried out to validate these anatomical parameters of dangerous ethmoid in clinical practice.

## CONCLUSIONS

The surgeon’s understanding of the anatomy of a patient’s ethmoid roof and its possible variations is crucial for countering possible complication risks during surgery. The most common occurrence of the ethmoid roof on the basis of keros classification is keros type II. The width of the olfactory fossa, middle turbinate length and medial orbital distance were higher in males and on the right side attention should be given for asymmetry.

## Conflict of Interest:

None.
